# Dyslipidemia in retinal metabolic disorders

**DOI:** 10.15252/emmm.201910473

**Published:** 2019-09-05

**Authors:** Zhongjie Fu, Chuck T Chen, Gael Cagnone, Emilie Heckel, Ye Sun, Bertan Cakir, Yohei Tomita, Shuo Huang, Qian Li, William Britton, Steve S Cho, Timothy S Kern, Ann Hellström, Jean‐Sébastien Joyal, Lois EH Smith

**Affiliations:** ^1^ Department of Ophthalmology Harvard Medical School Boston Children's Hospital Boston MA USA; ^2^ Manton Center for Orphan Disease Harvard Medical School Boston Children's Hospital Boston MA USA; ^3^ National Institute on Alcohol Abuse and Alcoholism National Institutes of Health Bethesda MD USA; ^4^ Department of Pediatrics, Pharmacology and Ophthalmology CHU Sainte‐Justine Research Center Université de Montréal Montreal QC Canada; ^5^ Department of Pharmacology and Therapeutics University of Montreal Montreal QC Canada; ^6^ Beijing Tongren Eye Center Beijing Tongren Hospital Capital Medical University Beijing China; ^7^ Center for Translational Vision Research Gavin Herbert Eye Institute Irvine CA USA; ^8^ Section for Ophthalmology Department of Clinical Neuroscience Institute of Neuroscience and Physiology Sahlgrenska Academy University of Gothenburg Göteborg Sweden

**Keywords:** dyslipidemia, FGF21, photoreceptor, retinal metabolism, β‐oxidation, Neuroscience, Vascular Biology & Angiogenesis

## Abstract

The light‐sensitive photoreceptors in the retina are extremely metabolically demanding and have the highest density of mitochondria of any cell in the body. Both physiological and pathological retinal vascular growth and regression are controlled by photoreceptor energy demands. It is critical to understand the energy demands of photoreceptors and fuel sources supplying them to understand neurovascular diseases. Retinas are very rich in lipids, which are continuously recycled as lipid‐rich photoreceptor outer segments are shed and reformed and dietary intake of lipids modulates retinal lipid composition. Lipids (as well as glucose) are fuel substrates for photoreceptor mitochondria. Dyslipidemia contributes to the development and progression of retinal dysfunction in many eye diseases. Here, we review photoreceptor energy demands with a focus on lipid metabolism in retinal neurovascular disorders.

**Glossary**
Autophagy: A process induced under stress to process cellular wastes to reduce toxicity and provide fuel for mitochondria.
Fatty acid β‐oxidation: The catabolic process that breaks down fatty acid molecules to generate acetyl‐CoA, which in mitochondria enters the citric acid cycle for energy (ATP) production. β‐oxidation occurs in the peroxisomes and mitochondria but in peroxisomes no ATP is produced.
Glycolysis: The process of breaking down glucose into pyruvic acid for energy production.
Neovascularization: Uncontrolled blood vessel growth in the eye. New vessels are often fragile and leaky, causing blindness in the late stage of neovascular retinal diseases.
Oxidative phosphorylation: The process to form ATP through the transfer of electrons from NADH or FADH2 to oxygen by a series of electron carriers in the mitochondrial membrane.
Photoreceptors: A retinal neuronal cell that is capable of visual phototransduction, converting light signals to electric signals. They possess the highest density of mitochondria in the body.
Retinal pigment epithelium: The pigmented cell layer provides nutrients and clears wastes for photoreceptors.

## Photoreceptor biology and retinal lipid use

### Energy demands of the retina

Vertebrate retinas are light‐sensitive neural tissues. Rod and cone photoreceptors of the retina utilize photosensitive pigments to convert photons into electrical impulses (phototransduction; Arshavsky *et al*, 2002). The retina uses more energy in the dark than in light to maintain the “dark current”. In the light, there is an ongoing outward potassium (K^+^) current through non‐gated K^+^‐selective channels, which induces sodium (Na^+^) ion channel closure and hyperpolarization of photoreceptors. In the light, glutamate release is suppressed and neurons are excited, leading to phototransduction.

By contrast, in the dark, perpetually open (Na^+^) channels allow a steady flow of ions into the cell, thereby resulting in cellular depolarization (dark current) and glutamate release, which inhibits photoreceptor excitation (Stryer, 1991). More than half of photoreceptor energy (adenosine triphosphate, ATP) is used by Na^+^/K^+^ ATPase ion pumps to maintain intracellular ion levels (Hagins *et al*, 1970; Okawa *et al*, 2008).

The replacement of shed photoreceptor outer segments is also energy intensive (Du *et al*, 2015; Ng *et al*, 2015). Photoreceptors maintain a consistent outer segment length by balancing disk shedding and assembly (Young, 1967; Young & Bok, 1969; LaVail, 1976). Continuous shedding of “used” outer segments containing lipids damaged by light and oxidation is critical for the maintenance of normal retinal function (Fliesler & Anderson, 1983) perhaps as a fuel source scavenged by retinal pigment epithelium (RPE). Other lipid fuel sources may be serum lipids processed by Müller glial cells, and lipids synthesized at a high rate in the inner segments (Wang *et al*, 2005; Kevany & Palczewski, 2010; Casson *et al*, 2013). The details of lipid processing in the retina are not fully defined; however, some lipids are used as fuel/energy sources while the others, which cannot be synthesized, are recycled (Chen & Anderson, 1993; Mukherjee *et al*, 2007).

### Fuel sources for the retina to make ATP

ATP, used to transfer energy, is generated via two metabolic pathways: glycolysis in the cytoplasm and oxidative phosphorylation (OXPHOS) in mitochondria. Glycolysis converts one glucose to two pyruvates (yielding 2 ATP). In the presence of oxygen, pyruvate is further converted to acetyl‐CoA, which enters the Krebs cycle (yielding 2 ATP) and forms electron donors for OXPHOS (yielding 34 ATP). However, when there is an oxygen shortage, pyruvate is converted into lactate. In the retina, even without oxygen deficits glucose is mostly metabolized though glycolysis rather than OXPHOS (called aerobic glycolysis or the Warburg effect). It has been shown that in pig retinal explants, only 20% of glucose is oxidized (Wang *et al*, 1997); despite access to oxygen, 80% is used for glycolysis, which provides intermediates for outer segment synthesis.

The remaining oxidation is of carbons derived from non‐glucose sources such as lipids (Joyal *et al*, 2016). In rat and rabbit retinas, there is no difference in lactate production in darkness and in light, indicating that aerobic glycolysis is not required for the energy needed to maintain the dark current (Cohen & Noell, 1960; Winkler, 1981). The dark current is maintained with OXPHOS, using over 40% of retinal oxygen (Ames *et al*, 1992). The fuel source for OXPHOS was unknown for almost 60 years, as most glucose clearly is not metabolized through OXPHOS (Cohen & Noell, 1960). Recently, lipid oxidation (palmitic acid C16:0) was found to be used for energy production in photoreceptor mitochondria (Joyal *et al*, 2016). The major fatty acids of physiological importance in energy metabolism are those with a chain length ≥ 16 carbons. Fatty acid β‐oxidation (OXPHOS) of one 16‐carbon fatty acid produces 129 ATP, while full oxidation of glucose produces 38 ATP. Knowledge of the potential impact of other fatty acids on retinal metabolism is still limited.

### Cones versus rods in energy consumption and production

Cones are more metabolically active than rods (Nikonov *et al*, 2006; Okawa *et al*, 2008). In darkness, cones and rods have comparable ATP expenditures, and similar dark currents. However, in the light, rod responses are suppressed, thereby reducing total retinal energy consumption by > 75%. But cones do not saturate in bright light so the energy demand remains high. Even when cones are maximally bleached, they still have a baseline need that is more than 50% of the dark current (Nikonov *et al*, 2006).

It is essential to coordinately control the synaptic terminal ATP production and Ca^2+^ concentration to regulate transient exocytosis and ensure recovery for the next action potential (Johnson *et al*, 2007). Cones increase ATP production by increasing the number of mitochondria (about twofold more than rods) and mitochondrial cristae surface membrane area (about threefold more than rods; Perkins *et al*, 2003). Cones lower Ca^2+^ levels during light adaption and increase their response kinetics by utilizing a low affinity/high turnover Na^+^‐Ca^2+^ exchanger, while rods use high affinity/low turnover plasma membrane Ca^2+^ ATPase (Johnson *et al*, 2007). The knowledge of fuel use in cones and rods is still limited. Loss of hexokinase 2 (a key aerobic glycolysis enzyme) in rods inhibits rod function but not rod survival, while cone hexokinase 2 loss does not affect photoreceptor function (Petit *et al*, 2018), suggesting that aerobic glycolysis is not necessary for photoreceptor survival, but is a metabolic choice to maintain neuronal function.

### Peroxisome fatty acid β‐oxidation

**Figure 1:**
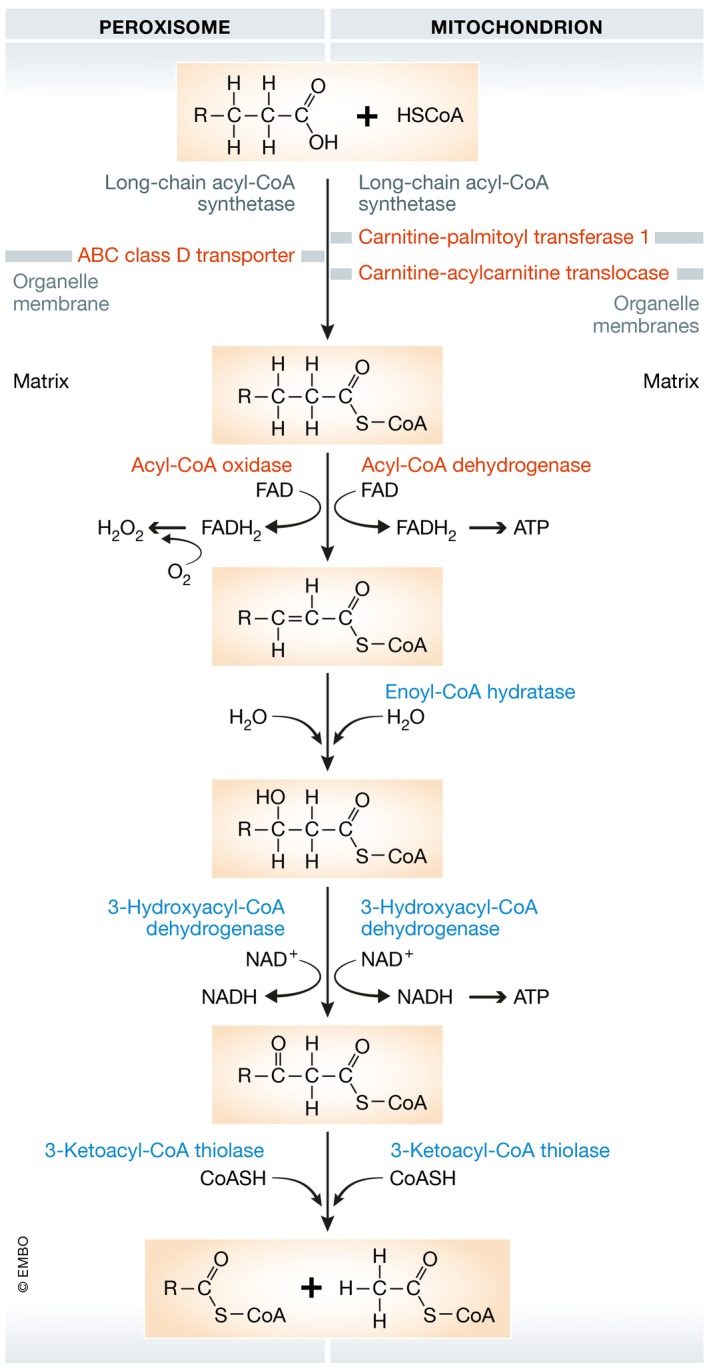
β‐oxidation pathway in peroxisome and mitochondria

Fatty acid degradation, through β‐oxidation, takes place in both mitochondria and peroxisomes in mammals. Peroxisomal degradation breaks down long‐chain fatty acids (producing no ATP), into shorter chain fatty acids that can be used by mitochondria for further oxidation to acetyl‐CoA, which enters the Krebs cycle to produce ATP (Fig 1; Poirier *et al*, 2006; Schrader *et al*, 2015).

Very long‐chain monocarboxylic (≥22 carbons) and long‐chain dicarboxylic fatty acids are oxidized only in peroxisomes (Poirier *et al*, 2006). In addition, polyunsaturated fatty acids are also oxidized faster in peroxisomes than in mitochondria (Hiltunen *et al*, 1986). Long‐chain fatty acids (13–21 carbons) must first be conjugated to either coenzyme A (peroxisomes) or carnitine (mitochondria) outside the organelle and then imported into organelles by ABC class D transporters (peroxisomes) or carnitine‐acylcarnitine translocases (mitochondria). Fatty acids are subsequently degraded by β‐oxidation, which involves four enzymes and leads to the release of acetyl‐CoA, FADH_2_, and NADH (Fig 1). Acetyl‐CoA enters the Krebs cycle where it is oxidized into CO_2_ and H_2_O, and generates additional FADH_2_ and NADH. FADH_2_ and NADH from β‐oxidation and the Krebs cycle are then used for ATP production by the mitochondrial electron transport chain.

### Retinal lipid composition

**Figure 2:**
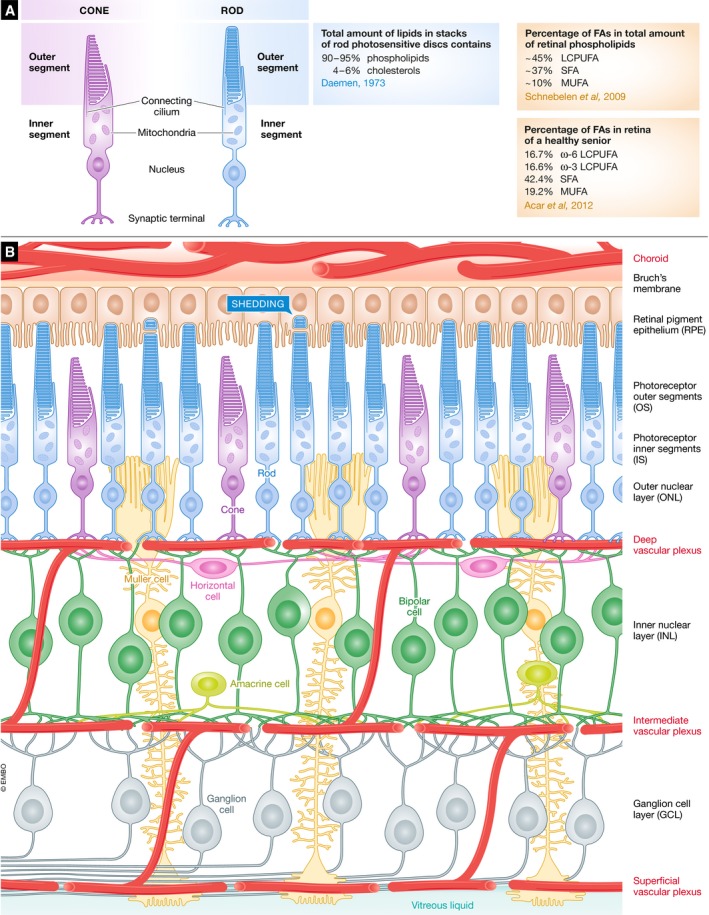
Schematics of photoreceptor and retinal structure (A) Schematics of rod and cone structure. (B) Schematics of retinal neuronal and vascular arrangement. RPE, retinal pigment epithelium; OS/IS, outer segments/inner segments; ONL, outer nuclear layer; INL, inner nuclear layer; GCL, ganglion cell layer.

A rod photoreceptor has three functional domains: (i) synaptic terminal, (ii) inner segment, and (iii) outer segment (Fig 2A). One retinal lipid source comes from shed photoreceptor outer segments. Rod outer segments consist of stacks of photosensitive disks, which contain proteins (predominantly photosensitive pigments) and lipids (Fliesler & Anderson, 1983), predominantly phospholipids (90–95% of total lipids) and cholesterols (4–6%) (Daemen, 1973).

Phospholipids consist of a phosphate “head” with fatty acid “tails”, which can be cleaved to provide fatty acids. Retinal phospholipids contain an abundance of long‐chain polyunsaturated fatty acid (LCPUFA, ~45% of total phospholipids), saturated fatty acid (SFA, ~37% of total phospholipids), and monounsaturated fatty acid (MUFA, ~10% of total phospholipids; Schnebelen *et al*, 2009). While fatty acid composition analysis in the human retina is limited, the retina of a healthy senior is composed of 16.7% ω‐6 LCPUFA, 16.6% ω‐3 LCPUFA, 42.4% SFA, and 19.2% MUFA (Acar *et al*, 2012).

During postnatal development, rod lipid composition transitions from rich in saturated fatty acids to rich in unsaturated fatty acids (Scott *et al*, 1988). The increasing unsaturated lipid portion of the maturing retina is biased for selective accretion of docosahexaenoic acid (DHA) while arachidonic acid (AA) levels are reduced (Alessandri & Goustard‐Langelier, 2001). Docosahexaenoic acid accounts for approximately 35% of total phospholipid FA in the retina and 50% in rod outer segments (Stinson *et al*, 1991), while AA accounts for approximately 8–10% of total phospholipid fatty acid in rod outer segments.

### Transcriptional control of retinal cell functions

**Figure 3:**
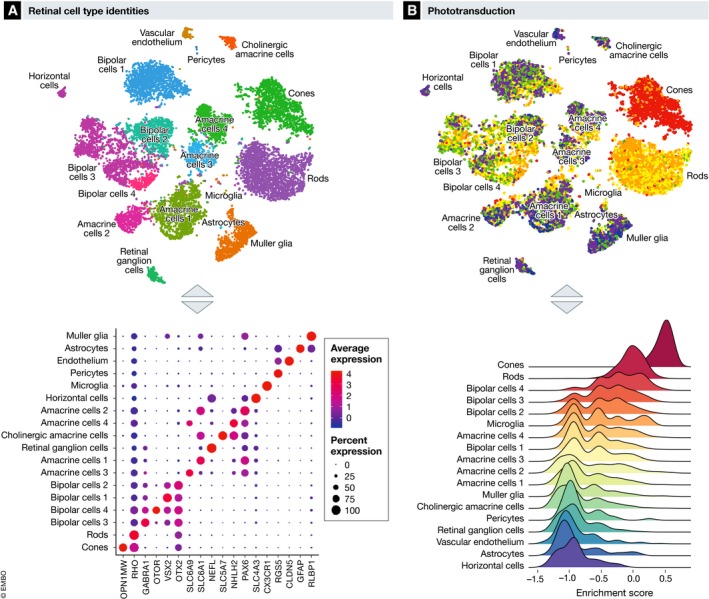
Cell‐specific transcriptional regulation of retinal functions Adapted from Macosko *et al*, Cell 2015. (A) Retinal cell types can be identified using single‐cell RNAseq based on cell‐specific expression of genes markers. OPN1MW, Opsin 1, medium wave sensitive; RHO, rhodopsin; GABRA1, gamma‐aminobutyric acid type A receptor alpha1 subunit; OTOR, otoraplin; VSX2, visual system homeobox 2; OTX2, orthodenticle homeobox 2; SLC6A9, solute carrier family 6 member 9; SLC6A1, solute carrier family 6 member 1; NEFL, neurofilament light; SLC5A7, solute carrier family 5 member 7; NHLH2, nescient helix‐loop‐helix 2; PAX6, paired box 6; SLC4A3, solute carrier family 4 member 3; CX3CR1, C‐X3‐C motif chemokine receptor 1; RGS5, regulator Of G protein signaling 5; CLDN5, claudin 5; GFAP, glial fibrillary acidic protein; RLBP1, retinaldehyde binding protein 1. (B) Transcriptomic enrichment for specific pathway such as the phototransduction pathways can be scored using gene set variation analysis based on highly variable genes between retinal cell types.

The retina contains more than 10 different cell types that contribute uniquely to phototransduction (Fig 2B), requiring a highly individualized gene expression pattern. The emergence of single‐cell transcriptomics (scRNAseq) provides insight into the metabolism of individual cells within the retina, which is likely to lead to a greater understanding of the cellular metabolic influences on neovascularization. scRNAseq examines the combinatorial expression of genes, which leads to the clustering of retinal cells according to their gene expression patterns (Fig 3A; Macosko *et al*, 2015). The scRNAseq approach is very efficient in discovering new retinal cell subtypes (Shekhar *et al*, 2016; Rheaume *et al*, 2018). Beside identity markers associated with specialized functions (like phototransduction in photoreceptors; Fig 3B), different retinal cells regulate specific metabolic genes at the transcriptional level to perform certain functions. However, caution is required when analyzing transcriptomic data from rod photoreceptors, as this cell type has low basal gene expression (Macosko *et al*, 2015), which is correlated with a uniquely closed chromatin architecture compared to cones (Hughes *et al*, 2017). Moreover, rods are very sensitive to single‐cell dissociation since the end part of rod outer segments is buried in the RPE and may be separated from the main cell body during retinal digestion. Single‐nucleus RNAseq may therefore be more suitable to assess the rod transcriptome *in situ* (Habib *et al*, 2017).

## Dyslipidemia in neurovascular retinopathies

Metabolic dysfunction and dyslipidemia produce deleterious effects on the eye (Folz & Trobe, 1991; Chang & Wu, 2013; Yonekawa *et al*, 2015). Dyslipidemia is characterized by an abnormal circulating lipid profile including triglycerides, cholesterol, low‐density lipoproteins (LDL), high‐density lipoproteins (HDL), or polyunsaturated fatty acids. In premature infants, high triglycerides are associated with increased severity of retinopathy of prematurity (ROP; Sinclair *et al*, 2018). The ω‐6 LCPUFA, arachidonic acid, level is also significantly lower in severe ROP in premature infants at postmenstrual age of 32 weeks (Lofqvist *et al*, 2018). Although the results from many studies exploring the associations between diabetic retinopathy (DR) and lipid abnormality are inconsistent, one study found that high circulating LDL cholesterol levels are a significant risk factor for diabetic macular edema and retinal hard exudates (Chang & Wu, 2013). In advanced age‐related macular degeneration (AMD), high HDL cholesterol levels are implicated in the disease pathogenesis in European and Asian populations (Cougnard‐Gregoire *et al*, 2014; Fan *et al*, 2017). A recent European Eye Epidemiology consortium study found that HDL is associated with an increased risk of AMD and drusen development, while triglycerides are associated with a decreased risk of AMD and drusen development. Variants in lipid genes and their association with cholesterol levels are unclear. The cholesteryl ester transfer protein risk variant (rs17231506) for AMD was associated with increased HDL cholesterol levels, but lipase C risk variants (rs2043085, rs2070895) were negatively linked with HDL cholesterol levels (Colijn *et al*, 2019). In retinitis pigmentosa (RP) versus control patients, decreased plasma ω‐3 and ω‐6 LCPUFA are found (Converse *et al*, 1983; Holman *et al*, 1994).

Dietary modulation of the lipid supply can positively influence diseases with pathological neovascularization such as ROP, AMD, and DR in patients and in animal models of retinopathy (Gong *et al*, 2017). Photoreceptor energy demands drive vessel growth (Sapieha, 2012; Joyal *et al*, 2016, 2018; Fu *et al*, 2018), while photoreceptor‐derived oxidative stress and inflammation lead to retinal vascular damage or regression (Kern & Berkowitz, 2015; Sun *et al*, 2017). Retinal disorders such as ROP, DR, AMD, RP, and Zellweger spectrum disorder (ZSSD) are associated with disturbances in photoreceptor activity, which may further affect the blood supply and induce pathological vascular remodeling during disease progression.

### Retinopathy of prematurity

Retinopathy of prematurity is a leading cause of blindness in children worldwide (Hellstrom *et al*, 2013). After preterm birth, the immature retinal vasculature growth is suppressed, secondary to oxygen supplementation, loss of growth factors provided *in utero*, and metabolic dysregulation. As the neural retina slowly matures, metabolic demand increases, particularly in photoreceptors. The relatively avascular retina becomes hypoxic and deprived of nutrients, driving vascular growth factor expression and subsequent neovascularization. The onset of neovascular ROP at ~32 weeks postmenstrual age coincides with the rapid development and increased metabolic demand of rods (Fulton *et al*, 1999; Hansen *et al*, 2017). This observation is supported by rodent studies. In mice, hyperglycemia (a key risk factor for ROP) triggers photoreceptor metabolic alterations and delays retinal vascular development (Fu *et al*, 2018). In rats, early photoreceptor dysfunction also predicts subsequent neovascularization (Akula *et al*, 2010).

In premature infants, there is a ~44% decrease in DHA after preterm birth, and serum DHA levels remain low for at least 4 weeks (Lapillonne & Jensen, 2009; Martin *et al*, 2011). Severe ROP is reduced in premature infants (GA < 32 weeks) receiving ω‐3 LCPUFA versus parenteral soybean and olive oil supplementation (Pawlik *et al*, 2014). There is also an association between low serum levels of ω‐6 LCPUFA (AA) and later development of ROP (Lofqvist *et al*, 2018). In mice, dietary ω‐3 versus ω‐6 LCPUFA suppresses retinal neovascularization (Connor *et al*, 2007; Fu *et al*, 2017b). Further studies on the impact of DHA and AA and other lipids on photoreceptor function and metabolism are needed.

### Diabetic retinopathy

In addition to ROP, DR is also associated with abnormal energy metabolism. DR, a significant complication of diabetes, starts with vascular loss (non‐proliferative DR), followed by neovascularization (proliferative DR). In DR, abnormalities in retinal neural responses occur early before vascular abnormalities are seen, suggesting that neuronal metabolic demands drive vessel growth (De Benedetto *et al*, 2014; Pescosolido *et al*, 2015). Mitochondrial dysfunction is accompanied by oxidative stress (Barot *et al*, 2011), which induces a wide range of microvascular abnormalities throughout the course of DR (Kowluru & Mishra, 2015). In diabetic mice, photoreceptors with their high density of mitochondria contribute to the majority of induced retinal oxidative stress and inflammation, which is associated with retinal vessel loss in DR (Du *et al*, 2013; Liu *et al*, 2016; Tonade *et al*, 2016).

There is clear evidence of neurovascular cross talk in DR. In patients with both proliferative DR and progressive photoreceptor degeneration (RP), spontaneous neovascular regression occurs when photoreceptor loss from RP becomes clinically evident (Lahdenranta *et al*, 2001). As there is higher retinal energy consumption in darkness versus light, illuminating the retina with 507‐nm light during sleep might reduce the risk of DR progression (Sivaprasad & Arden, 2016). In fact, exposure to a 505‐nm light during sleep leads to the regression of macular edema and improved visual function in early DR patients (Arden *et al*, 2011). However, a recent multi‐year phase 3 clinical trial (CLEOPATRA) of wearing a light mask at night in DR patients failed to support the hypothesis that decreasing energy needs for photoreceptor “dark current” would inhibit diabetic macular edema (Sivaprasad *et al*, 2018).

Dyslipidemia is associated with more retinal abnormalities and faster progression of DR (Sacks *et al*, 2014; Hammer & Busik, 2017). Increasing dietary PUFA versus saturated FA is associated with a reduced incidence and severity of DR (Sasaki *et al*, 2015). A Mediterranean diet with olive oil or nut supplements showed an additional 48% decrease in incidence of DR in type 2 diabetes when the diet also included ≥ 500 mg/day DHA plus eicosapentaenoic acids, or at least 2 weekly servings of oily fish (Sala‐Vila *et al*, 2016). In murine models of early DR, fish oil and a ω‐3 LCPUFA‐enriched diet preserve retinal neuronal function (Yee *et al*, 2010; Sapieha *et al*, 2012). Linoleic acid‐ versus saturated‐fat‐rich diets inhibit progression of diabetic microangiopathy (Houtsmuller *et al*, 1980). Not all lipids appear to affect DR. The absence of acid sphingomyelinase (ASM) in ASM^−/−^ mice or inhibition of ASM activity by DHA inhibited the diabetes‐induced degeneration of retinal capillaries (Opreanu *et al*, 2011). Studies show no association between total cholesterol or high‐density lipoprotein and incidence of DR or macular edema in long‐term type 1 diabetes (Klein *et al*, 2015). Taken together, these findings suggest a link between some aspects of dyslipidemia and DR progression. As such, dietary modulation of specific lipids may help prevent or treat DR.

### Age‐related macular degeneration

The human macula, critical for central vision, consists of a small cone‐dominated fovea surrounded by a rod‐dominated parafovea. AMD particularly affects the macula and is the leading cause of legal blindness in the elderly. Clinically, AMD is classified as either dry or wet (neovascular) based on the absence or presence of pathologic blood vessels invading into the photoreceptor layer (Ambati & Fowler, 2012). Current treatments mainly target neovascular AMD; no drugs are approved by the U.S. Food and Drug Administration for dry AMD.

**Figure 4:**
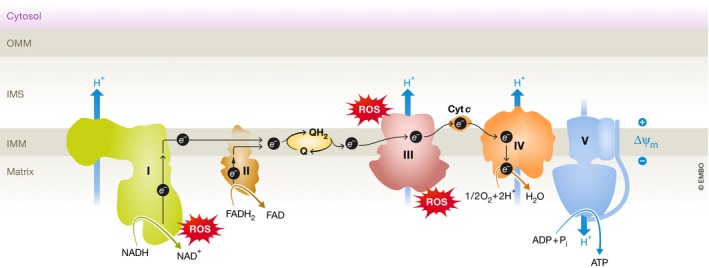
Schematics of electron transport chain (ETC) The ETC passes electrons from NADH and FADH_2_ to protein complexes (I to V) and mobile electron carriers coenzyme Q (CoQ) and cytochrome *c* (Cyt *c*). Oxygen (O_2_) is the final electron recipient. The transfer of electrons generates energy to pump protons (H^+^) from the mitochondrial matrix into the intermembrane space. An electrochemical proton gradient is created across the inner mitochondrial membrane, allowing the protons to pass through complex V (ATP synthase) to generate adenosine triphosphate (ATP) from adenosine diphosphate (ADP). Complex I, NADH coenzyme Q reductase, complex II, succinate dehydrogenase, complex III, cytochrome *bc*_*1*_ complex, complex IV, cytochrome *c* oxidase. Complex I and complex III are the main sites for superoxide (ROS) formation.

Mitochondrial morphological changes and dysfunction occur in degenerating macular cones and likely contribute to AMD progression (Barron *et al*, 2001; Litts *et al*, 2015). Mitochondrial abnormalities can cause overproduction of superoxide radicals, primarily in the electron transport chain (Fig 4; Selivanov *et al*, 2011). Oxidative stress causes damage to cell structures, lipids, proteins, and DNA, and particularly affects metabolically active neuronal cells, like photoreceptors. Photoreceptor loss is seen in AMD donor eyes (Curcio *et al*, 1996). In aging and in early AMD, there is a prominent decline in rod‐mediated ERG sensitivity (Jackson *et al*, 2002). Additionally, cone dysfunction can predict early AMD and is a reliable measure of AMD progression (Hogg & Chakravarthy, 2006).

As well as mitochondrial dysfunction, dyslipidemia is also implicated in dry AMD pathogenesis (Gong *et al*, 2017). The most established clinical hallmark of dry AMD is the formation of subretinal drusen, extracellular deposits rich in lipid and protein (Hageman *et al*, 2001). Although the exact mechanism of drusen formation is unclear, Bruch's membrane, found between the choriocapillaris and RPE, thickens due to accumulation of oxidized lipids, lipid‐related molecules, and inflammatory debris preceding drusen formation. This slows down nutrient and waste transportation between RPE cells and choroidal vessels, leading to malfunction of RPE cells (Sarks *et al*, 2007; Curcio, 2018a,b).

Epidemiologic studies link increased HDL levels with AMD across different populations (Fan *et al*, 2017; Colijn *et al*, 2019). Genome‐wide association studies also identify several HDL cholesterol genes associated with AMD susceptibility, including genes encoding ATP‐binding cassette transporter A1 (*ABCA1*)*,* cholesteryl ester transfer protein (*CETP*)*,* apolipoprotein E (*APOE*), hepatic lipase C (*LIPC*), and lipoprotein lipase precursor *(LPL)* (Chen *et al*, 2010; Neale *et al*, 2010; Fritsche *et al*, 2013, 2016). Impaired ABCA1‐mediated cholesterol efflux in mouse RPE cells or subretinal macrophages induces lipid accumulation and retinal degeneration (Lyssenko *et al*, 2018; Storti *et al*, 2019). ApoE is important for the transport of lipids across cell membranes and is highly expressed by RPE cells. ApoE‐null mice exhibit raised serum triglycerides and cholesterol. Thickened Bruch's membrane, and accumulation of lipid deposits in the basal RPE and Bruch's membrane are seen in ApoE‐null and genetically engineered ApoE‐mutated mice (Malek *et al*, 2005; Edwards & Malek, 2007). However, how CETP, LIPC, and LPL link to AMD pathogenesis is still unclear.

High plasma levels of high‐density lipoprotein cholesterol are associated with an increased risk for advanced AMD (Fan *et al*, 2017). A 42% decreased incidence of AMD is associated with high plasma ω‐3 LCPUFA levels in a large cohort study of US female health professionals (Christen *et al*, 2011). A 30% decrease in central geographic atrophy development and a 50% decrease in neovascular AMD development are found in participants with high versus low ω‐3 LCPUFA intake in the Age‐Related Eye Disease Study (AREDS) (Sangiovanni *et al*, 2009). There was no further reduced risk of progression to advanced AMD in participants with ω‐3 LCPUFA supplementation in the AREDS2 study; however, the participants had a much higher baseline level of circulating ω‐3 LCPUFA in comparison with those in the first AREDS (Souied *et al*, 2015). It also may be that other fats in fish (alone or in combination with DHA) are required to suppress AMD progression. In a Japanese population with a high baseline intake of fish oil, there was no significant association between serum ω‐3 LCPUFA levels and AMD progression (Kabasawa *et al*, 2011). Dietary ω‐3 LCPUFA inhibits neovascularization in a laser‐induced wet AMD mouse model and in mice lacking the very low‐density lipoprotein receptor (VLDLR) (Fu *et al*, 2017b). The lack of VLDLR promotes the development of neovascularization originating from the superficial retinal vasculature similar to some neovascularization seen in AMD. The lack of VLDLR leads to intracellular lipid and glucose insufficiency which drives neovascularization, including retinal angiomatous proliferation and choroidal neovascularization (Joyal *et al*, 2016). Therefore, both clinical and experimental investigations support the concept that dyslipidemia may be associated with AMD progression.

### Retinitis pigmentosa

Retinitis pigmentosa is also associated with abnormal energy metabolism. There are ~60 genes (to date), mostly expressed in rods, which are involved in RP retinal degenerations (Ali *et al*, 2017). In RP, the initial loss of rods results in night blindness and loss of peripheral vision; central (cone) vision is initially preserved but eventually central vision is also lost, secondary to a bystander effect (Punzo *et al*, 2009; Ait‐Ali *et al*, 2015). In mouse models of RP, 34.9% of gene expression changes following cone loss are associated with cellular metabolism (Punzo *et al*, 2009), suggesting that improving fuel sources (perhaps such as lipids for FA β‐oxidation) may improve cone metabolism. Mathematical models predict that preventing a 1–2% decrease in nutrients can permanently halt cone death even when 90% have already died (Camacho *et al*, 2016). Therefore, improving nutrient availability is a reasonable general approach to increase cone survival in RP. In RP patients, reduced ocular blood flow is also described as possibly associated with a decreased neuronal demand for nutrient supply (Falsini *et al*, 2011). Further studies are needed to establish the link between retinal vascular changes at different stages of RP.

### Zellweger syndrome spectrum disorders

Zellweger syndrome spectrum disorders (ZSSD), including Zellweger syndrome, neonatal adrenoleukodystrophy, and infantile Refsum disease (Smith *et al*, 2016), are caused by defects in any of the peroxisomal PEX genes (Crane, 2014), resulting in peroxisomal lipid metabolic dysfunction. As peroxisomes break down long‐chain fatty acids to shorter length chains that can be used in mitochondria, deficits in PEX genes often also result in mitochondrial dysfunction. In ZSSD, the central nervous system is severely affected (Vamecq *et al*, 2014), producing unique ocular deficits including pigmentary retinopathy and optic atrophy, corneal opacification, cataract, and glaucoma (Folz & Trobe, 1991). In addition, attenuated retinal vasculature and macular edema are reported in infantile Refsum disease (Pakzad‐Vaezi & Maberley, 2014).

Mitochondrial perturbation rapidly occurs following the loss of functional peroxisomes (Salpietro *et al*, 2015; Schrader *et al*, 2015). Very long‐chain fatty acids (VLCFAs) accumulate in ZSSD; however, DHA (22:6ω3) is reduced in the plasma and brain (Poulos *et al*, 1986; Harding *et al*, 1999). Docosahexaenoic acid treatment maintains visual acuity and retinal function in patients with peroxisome biogenesis disorders (Noguer & Martinez, 2010). Over‐accumulation of VLCFAs is also found in mouse models of peroxisomal biogenesis defects (Baes, 2000; Baes & Van Veldhoven, 2012). VLCFAs may affect membrane properties (Sassa & Kihara, 2014), and defects in the breakdown of VLCFAs may also cause substrate shortage for mitochondrial fatty acid β‐oxidation.

### Pathways modifying metabolic lipid use

Peroxisome proliferator‐activated receptor‐alpha (PPARα) and PPARγ are nuclear receptors involved in modulating lipid metabolic homeostasis. PPARα controls lipoprotein lipase expression and triglyceride metabolism, while PPARγ upregulates enzymes involved in steps of fatty acid metabolism like fatty acid entry into mitochondria and peroxisome (Gervois *et al*, 2000). Genetic deficiency of PPARα in mice leads to a decrease in lipid transporters and retinal degeneration (Pearsall *et al*, 2017). PPARγ is required for ω‐3 LCPUFA‐induced attenuation of mouse retinal neovascularization (Stahl *et al*, 2010). PPARγ coactivator‐α (PGC‐1α) regulates mitochondrial biogenesis and respiration (Alaynick, 2008). High‐fat diet‐exposed mice are more likely to develop AMD‐like phenotypes with lack of PGC‐1α (Zhang *et al*, 2018). PGC‐1α activation increases RPE metabolism and protects against oxidative damage (Satish *et al*, 2018). Therefore, PPARα, PPARγ, and PGC‐1α may modulate retinal lipid metabolism and therefore be pathways to manipulate in disease treatment.

Cyclooxygenases (COX), lipoxygenases (LOX), and cytochromes P450 (CYP)‐mediated LCPUFA metabolism is important in regulating ocular inflammation, particularly through the LOX and CYP pathways (Gong *et al*, 2017). Inhibiting COX does not affect proliferative retinopathy (Sapieha *et al*, 2011). LOX ω‐3 LCPUFA metabolites show anti‐inflammatory and anti‐angiogenic effects, while LOX ω‐6 LCPUFA metabolites are pro‐inflammatory and pro‐angiogenic (Sapieha *et al*, 2011). However, both CYP2C8 ω‐3 and ω‐6 LCPUFA metabolites are pro‐angiogenic and inhibition of CYP2C8 decreases ocular neovascularization (Shao *et al*, 2014; Gong *et al*, 2016a,b).

### Genetic association with retinopathies

Understanding genetic susceptibility to ocular disorders may help understand disease mechanisms. In premature infants, gene mutations in vascular endothelial growth factor (*VEGF*) and insulin growth factor 1 (*IGF1*) are associated with advanced ROP (Holmstrom *et al*, 2007). The association of VEGF and IGF1 with ROP is further identified clinically and in animal models (Hellstrom *et al*, 2013). Genetics in DR has been widely explored. Mutations in metabolic genes such as aldose reductase, endothelial nitric oxide synthase (eNOS), receptor for advanced glycosylation end product (RAGE), adiponectin, peroxisome proliferator‐activated receptor α and γ, and superoxide dismutase 2 (MnSOD), growth factors like *VEGF,* and erythropoietin (*EPO*), as well as inflammatory factors like complement factor H (*CFH*) and *CFB*, interleukin 6 and interleukin 10, have a positive association with DR (Hampton *et al*, 2015). Genome‐wide association studies for diabetic macular edema identify a new associate single nucleotide polymorphism in rs1990145 on chromosome 2 (within the second intron of the mitochondrial ribosomal protein L19, *MRPL19*; Graham *et al*, 2018). The function of *MRPL19* is unclear but other MRP genes including MRPL9 and MRPL23 are associated with retinitis pigmentosa (Kenmochi *et al*, 2001). Increasing evidence suggests a potential role of noncoding RNAs in regulating retinal inflammation during DR development (Gong & Su, 2017).

Delayed rod‐mediated dark adaption, the first functional biomarker for early AMD, is observed for both the age‐related maculopathy susceptibility 2 *(ARMS2)* A69S variant and the *CFH* Y402H variant in AMD patients. In healthy participants with normal macular function, the *ARMS2* A69S variant also was associated with delayed rod‐mediated dark adaption (Mullins *et al*, 2019). In three population‐based studies, the Rotterdam Study, the Beaver Dam Eye Study, and the Blue Mountains Eye Study, single nucleotide polymorphisms in the genes *ARMS2*,* CFH*, and complement factor H‐related 5 *(CFHR5)* significantly increase the risk of late AMD (Buitendijk *et al*, 2013). In AMD patients with *CFH* and *ARMS2* risk alleles, the treatment response to antioxidants is compromised (Awh *et al*, 2015). The *CFH* Y402H variant also seems to limit the effect of dietary DHA supplementation on CNV (Merle *et al*, 2015).

## Potential therapeutic targets

Anti‐vascular endothelial growth factor (VEGF) agents are the primary treatment for pathological retinal vessel growth in eye diseases (Klufas & Chan, 2015; Bakri *et al*, 2019). However, anti‐VEGF treatment is not always effective (Lux *et al*, 2007; Nigam *et al*, 2008) and may remain in systemic circulation up to a few months after a single intravitreal injection (Moorthy & Cheung, 2009; Ueta *et al*, 2009; Hapani *et al*, 2010; Bressler *et al*, 2012; Jalali *et al*, 2013; Avery *et al*, 2014). VEGF is an important growth factor to neurons and blood vessels. Therefore, inhibition of VEGF may affect normal neurovascular function.

Since photoreceptor metabolic needs drive neovascularization, improved retinal lipid metabolism might be another strategy to prevent or treat neurovascular retinal diseases. Increasing lipid β‐oxidation by hormonal and transcriptional factor regulation, as well as dietary intervention, may protect retinal function and decrease the demand for neovessels. Targeting dysmetabolism‐induced inflammatory responses may also suppress neovascularization.

### Fibroblast growth factor (FGF21), lipid metabolism, and autophagy

Since modulation of retinal metabolism may help restore energy homeostasis to prevent signaling for blood vessel recruitment and therefore prevent neovascularization, the end cause of neurovascular diseases, it is important to assess potential interventions that increase glucose uptake or increase fatty acid oxidation to improve energy homeostasis. A novel candidate for improved lipid metabolism is FGF21. FGF21 is a key metabolic regulator of lipid and glucose use (Kharitonenkov & Larsen, 2011; Lin *et al*, 2012; Markan *et al*, 2014). In type 2 diabetes, FGF21 decreases body weight and improves the lipid profile (Gaich *et al*, 2013; Talukdar *et al*, 2016). In obese type 2 diabetic mice, FGF21 lowers plasma triglycerides by lipoprotein catabolism in adipose tissue and maintains adipocyte phospholipid homeostasis (Foltz *et al*, 2012; Schlein *et al*, 2016; Ye *et al*, 2016; Stanislaus *et al*, 2017). FGF21 also increases lipid use in response to amino acid starvation (De Sousa‐Coelho *et al*, 2013). FGF21 functions through modulating the activities of PPAR and PGC‐1α. FGF21 is crucial for PPARα agonists to ameliorate metabolic disorders in obese mice (Goto *et al*, 2017). FGF21 regulates PPARγ activity and controls body fat (Dutchak *et al*, 2012). FGF21 also induces PGC‐1α to modulate glucose and fatty acid metabolism during starvation (Potthoff *et al*, 2009).

In diabetic mice with insulin deficiency, FGF21 enhances retinal antioxidant defense systems, reduces pro‐inflammatory cytokines, restores disorganized cone photoreceptor segments, and improves retinal function (Fu *et al*, 2018). FGF21 also regulates adiponectin (APN) production and secretion, and APN is key in mediating FGF21 modulation of glucose and lipid metabolism in mice (Holland *et al*, 2013; Lin *et al*, 2013). FGF21, mediated by APN which is associated with a number of metabolic retinal disorders (Fu *et al*, 2016), inhibits ocular neovascularization in mice (Fu *et al*, 2017a). FGF21 also increases APN secretion to diminish accumulation of ceramides in obese animals (Holland *et al*, 2013). Ceramide contributes to the development of DR and thus, modulating ceramide pathway may protect against DR progression (Fox *et al*, 2006; Opreanu *et al*, 2011).

Autophagy is induced in under stress (nutrient starvation, infection, or excess reactive oxygen species and recycles cytosolic components to remove damaged and dysfunctional cellular material to maintain cellular homeostasis, provide fuel, and recycle building blocks. In the retina, autophagy‐related proteins are mostly located in cellular layers that are rich in mitochondria and have high energy needs (Mitter *et al*, 2012). In addition, autophagy also plays an important role in phototransduction and rod integrity (Rodriguez‐Muela *et al*, 2012; Zhou *et al*, 2015). Aged mice with mutated autophagy genes have AMD‐like RPE defects (Zhang *et al*, 2017). Autophagy defects are also reported in human cells from AMD patients (Golestaneh *et al*, 2017). Some of the defects associated with autophagy deficiency are lipofuscin accumulation, reduced mitochondrial activity, and higher levels of reactive oxygen species—all of which affect angiogenesis. FGF21 influences autophagy. In mice, FGF21 is induced in neurons with mitochondrial dysfunction (Restelli *et al*, 2018). FGF21, induced with fasting, dephosphorylates transcription factor EB to induce genes involved in autophagy and lipid metabolism (Chen *et al*, 2017). In monosodium L‐glutamate‐induced obese mice, modeling nonalcoholic fatty liver disease, FGF21 induces autophagy to correct metabolic parameters (decreases triglycerides, improves insulin sensitivity) (Zhu *et al*, 2016). Further exploration of FGF21 in retinal lipid metabolism and autophagy is of great interest to evaluate its impact on retinal neurovascular stability.

### Fenofibrate (PPARα agonist and CYP2C antagonist)

Fenofibrate, a PPARα agonist, increases fatty acid β‐oxidation and improves mitochondrial function. Deficiency of PPARα leads to shortage of retinal energy production and neurodegeneration (Pearsall *et al*, 2017). In two large‐scale clinical trials, fenofibrate prevents the progression of DR. In the FIELD study, fenofibrate was found to reduce the need for laser‐treatment of DR in type 2 diabetes patients by ~30% (Keech *et al*, 2007). In the ACCORD Eye study, fenofibrate was found to reduce DR progression by ~40% (ACCORD Study Group *et al*, 2010). Fenofibrate prevents pathological neovascularization in the rat OIR model by suppressing hypoxia‐inducible factor and VEGF (Chen *et al*, 2013). Fenofibrate also reduces retinal vascular leakage in a murine diabetic model (Chen *et al*, 2013). Fenofibrate administration reduces retinal vascular leakage and downregulates VEGF production in the mouse model of type 1 diabetes (Chen *et al*, 2013). Fenofibrate is also a CYP2C antagonist (Schoonjans *et al*, 1996; Walsky *et al*, 2005). CYP2C metabolites from ω‐3 and ω‐6 LCPUFA show pro‐angiogenic effects in mouse models of ROP and AMD (Shao *et al*, 2014; Gong *et al*, 2016a). Inhibition of CYP2C with fenofibrate decreases retinal neovascularization (Gong *et al*, 2016b). Therefore, fenofibrate is a potential candidate to treat neurovascular defects in retinal metabolic disorders.

### Dietary ω‐3 LCPUFA intervention

The essential ω‐3 LCPUFA, DHA, influences neovascularization in retinopathy both in human patients and animal models. In AMD, increasing fish intake or ω‐3 LCPUFA supplementation (DHA, EPA) is associated with a decreased risk of AMD progression (Tan *et al*, 2009; Christen *et al*, 2011; Pinazo‐Duran *et al*, 2014). In premature infants and diabetic patients, plasma ω‐3 LCPUFA levels correlate with circulating APN and dietary intake of ω‐3 LCPUFA modulates circulating APN levels (Ito *et al*, 2014; Fu *et al*, 2015). In type 2 diabetic patients on a “healthy” Mediterranean diet, additional dietary intake of fish is associated with a 48% decreased incidence of proliferative DR (Sala‐Vila *et al*, 2016). In mouse models of proliferative ROP, DR, and AMD, dietary ω‐3 LCPUFA, mediated by APN, inhibits ocular neovascularization (Fu *et al*, 2015, 2017b).

### Free fatty acid receptor 1 (FFAR1)

FFAR1, which is activated by medium‐ and long‐chain fatty acids (Briscoe *et al*, 2003), governs glucose transport by regulating the expression of retinal GLUT1 (Joyal *et al*, 2016). In mice lacking VLDLR, a genetic deficiency in FFAR1 decreases retinal neovascularization, while FFAR agonist increases retinal neovascularization (Joyal *et al*, 2016). FFAR1 also mediates actions of nonenzymatically generated nitro‐oxidative products, transarachidonic acids, and induces cerebral microvascular degeneration in rats (Honore *et al*, 2013). Targeting FFAR1 may prevent pathologic endothelial cell proliferation and degeneration.

## Conclusions and perspectives

Generally, photoreceptor metabolism controls retinal neuronal and vascular development. Therefore, maintaining normal photoreceptor function will likely improve retinal vascular abnormalities in disease. As dyslipidemia contributes to disease progression in many retinal metabolic disorders, we may improve photoreceptor energy production by regulating lipid use and increasing lipid fuel sources, including those generated from autophagy. Targeting lipid metabolic modulation may improve neurovascular retinal function and decrease neovascularization.

### Conflict of interest

The authors declare that they have no conflict of interest.

**Pending issues**

• Anti‐VEGFA therapy affects neuronal function and has systematic effects as VEGFA is an important factor to promote neuronal function.
• Elucidate the link between photoreceptors and retinal vascular function.
• Identify the type of lipids as neuronal energy fuel sources.
• Explore potential therapeutic targets modulating lipid sensors.
